# The College of Nursing (Limited by Guarantee)

**Published:** 1921-01-08

**Authors:** 


### The College of Nursing (Limited by Guarantee).

YORKSHIRE CENTRE, LEEDS.

Friday, January 21.—Members' meeting at the Hospital for Women and Children, Leeds. Dancing 7.30 to 11.30 p.m. There will be cards for those not wishing to dance. Members able to be present are asked to communicate with Miss Lindall, Hon. Secretary, Hospital for Women and Children, before January 17.

